# Vanishing Point Extraction and Refinement for Robust Camera Calibration

**DOI:** 10.3390/s18010063

**Published:** 2017-12-27

**Authors:** Huan Chang, Fuan Tsai

**Affiliations:** 1 Department of Civil Engineering, National Central University, Taoyuan City 32001, Taiwan; 1984chang@gmail.com; 2 Center for Space and Remote Sensing Research, National Central University, Taoyuan City 32001, Taiwan

**Keywords:** vanishing points, camera calibration, visual geometry, pose estimation

## Abstract

This paper describes a flexible camera calibration method using refined vanishing points without prior information. Vanishing points are estimated from human-made features like parallel lines and repeated patterns. With the vanishing points extracted from the three mutually orthogonal directions, the interior and exterior orientation parameters can be further calculated using collinearity condition equations. A vanishing point refinement process is proposed to reduce the uncertainty caused by vanishing point localization errors. The fine-tuning algorithm is based on the divergence of grouped feature points projected onto the reference plane, minimizing the standard deviation of each of the grouped collinear points with an O(1) computational complexity. This paper also presents an automated vanishing point estimation approach based on the cascade Hough transform. The experiment results indicate that the vanishing point refinement process can significantly improve camera calibration parameters and the root mean square error (RMSE) of the constructed 3D model can be reduced by about 30%.

## 1. Introduction

Camera calibration is an important step in both photogrammetry and computer vision in order to extract metric information from two-dimensional (2D) images. Calibration includes interior orientation parameters (IOPs), e.g., focal length, principal point, lens distortion, skew, and aspect ratio, as well as exterior orientation parameters (EOPs), e.g., camera orientation and position. Various camera calibration methods have been developed using three-dimensional (3D) reference objects, 2D planes, or even lines [1]. Traditional camera calibration can be achieved using Tsai’s camera calibration model [2] or planar patterns [3]. This strategy only requires a known, planar calibration grid to estimate the IOPs and EOPs of the camera. Sturm and Maybank [4] summarized the singularities of calibration from one viewpoint with one or two planes. However, the calibration grid may not be easy to find and place properly, especially for in situ measurements.

Using vanishing points is an efficient process to obtain the camera pose directly in the scene by extracting parallel and perpendicular lines [5]. The geometric property of vanishing points has been well-defined in much of the photogrammetry literature. The principal point of the camera coincides with the orthocenter of the triangle, whose vertices are the three vanishing points for three orthogonal directions [6]. These lines commonly appear in man-made structures, for instance, rectangular windows, floor lines, columns, and beams, and are useful for detecting vanishing points. However, how to calculate precise and accurate vanishing points is a great challenge because any deviation will cause error propagation to camera calibration and subsequent object reconstruction processes [7].

This study developed an effective and flexible camera calibration method, which is particularly useful for on-site calibration, based on vanishing point extraction and refinement. The geometric relations between vanishing points and the camera system are defined according to collinearity condition equations. The developed algorithms require no prior camera parameters, nor internal or external parameters for onsite calibration. The proposed vanishing point refinement algorithm can reduce the uncertainty of vanishing point localization errors.

## 2. Vanishing Point Estimation

Projecting detected line segments in the image plane onto the Gaussian sphere is one of the classic approaches [8,9] for detecting vanishing points. Each line can be represented as a circle using angular parameterization (azimuth and elevation) of the Gaussian sphere. Vanishing points appear as the intersections of these circles, which represent the high occurrence rate of a particular element. Thales’ theorem can optimize the position of reference points [10] to overcome the projection center location problem in the Gaussian sphere. The Thales’ circle ensures that any line segment passing through the vanishing point must be perpendicular to the principal point in an isosceles triangle. The optimal triangle area minimization can then be achieved using least-squares techniques, and the accuracy and automation can be improved using random sample consensus (RANSAC) [11,12,13]. The Hough transform is a well-known method for detecting parametrical structures in images [14]. A double-cascaded Hough transform approach was introduced [15] to overcome the intrinsic limitations that prevent the extraction of the line segments along the main directions. In order to reduce the error and identify the possible points of intersection, a voting scheme was proposed based on a set of rules that weights each pair of intersected line segments in relation to their geometric characteristics [16].

An iterated Hough transform method was proposed to help find vanishing points and lines [17,18]. The method investigated a bounded slope–intercept parametric representation by splitting the original unbounded space into three bounded subspaces in order to keep the symmetry intact. It also employed a filtering algorithm before applying the second Hough transform to help extract important information emerging in each Hough space. Also based on the cascade Hough transform, a filtering and validation algorithm was implemented to cluster the line segments and estimate the vanishing points simultaneously [19,20].

Instead of using double transformation, another approach was to work directly on the first Hough polar plane [21]. By searching for a sinusoidal curve with appropriate amplitude and phase parameters, the least-squares minimization was applied with a weighting ratio. The ratio considered the number of times that the parameter set was observed as a mapping point of a line in the image. Similarly, Cantoni et al. [22] applied a filtering algorithm directly on the image plane after the first Hough transformation. This threshold-based filter works efficiently on edge-detected images, but the camera must be perpendicular to the reference plane and the horizontal line (vanishing line) should be parallel to the X axis. Some researchers combined fuzzy clustering algorithms to separate an image into several regions [23]. For each region, vanishing lines and the vanishing point can be located using the Hough-based method individually. This can help extract local vanishing points from specific objects.

Besides using transformation-based parameter estimation, the grouping together of features that satisfy a geometric relationship can also be used to detect and estimate vanishing points and lines. For example, McLean and Kotturi [24] integrated image processing and analysis algorithms to produce a method for practical feature extraction. In their method, the use of histogram analysis, clustering, and numerical optimization to locate vanishing points eliminates the need for any a priori estimates of the number or location of vanishing points. In addition, including a line quality measure allows large line data sets to be used without decreasing the overall quality of the vanishing point estimates, further increasing the degree of automation.

There are three common types of geometric grouping [25], which are: (1) a family of equally spaced coplanar parallel lines; (2) a planar pattern obtained by repeating some elements translating in the plane; and (3) a set of elements arranged in a regular planar grid. The presence or absence of geometric constraints is strong evidence for or against hypotheses such as parallelism in the real world. Almansa et al. [26] developed a detection algorithm that deduced the Gaussian sphere from the Helmoltz principle proposed by [27]. They divided the image plane into radial vanishing regions, and used minimum description length to restrict the number of false vanishing points. However, this approach works only when the vanishing point is not located within the image boundary. The direct measurement of the raw image can be simplified [28] using a RANSAC line model and expectation maximization (EM) [29], and the J-linkage clustering algorithm [30]. Nonetheless, lens distortion and strong image noise still degrade the performance of the line extraction and grouping process.

For real-time vanishing point detection, the local dominant orientation signature (LDOS) descriptor was introduced [31] to extract structural features directly from the image domain. The descriptor divides an image into several square blocks and accumulates the edge magnitude for each of them. The candidate vanishing blocks can be estimated by comparing the spatial distances from neighboring blocks containing the perspective lines with a similar direction (orientation).

## 3. Proposed Method for Vanishing Point Estimation

The proposed vanishing point estimation method consists of three parts. They are: image pre-processing, feature detection, and vanishing point localization.

### 3.1. Image Pre-Processing

The objective of pre-processing is to extract enough line segments for initial vanishing point detection. It is also useful for line-based radial distortion correction [32]. Firstly, straight line segments with sub-pixel accuracy are extracted using the Canny edge detector [33] with an additional linking and merging process. Merging aligned edges by orthogonal regression can increase the accuracy of their location and orientation.

**Figure 1:**
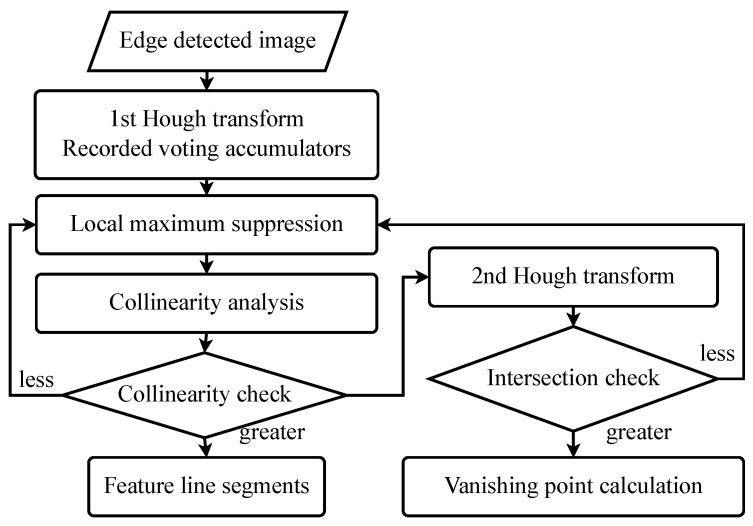
The procedure of initial vanishing point detection using double Hough transformation.

An improved cascade Hough transformation approach is proposed to extract line segments from the edge pixels and to classify them to the probable vanishing point candidates. The two steps of Hough transform are illustrated in Figure 1. The first Hough transform extracts line segments from the edge pixels. The initial vanishing point localization is processed using the output from the first Hough transform to group the line segments passing through the same region on the image. The details of the two Hough transforms are described in Section 3.2 and Section 3.3, respectively.

### 3.2. Feature Line Detection

The first Hough transform is commonly used to detect line segments in the image by keeping the dominant peaks in the normal-distance and normal-angle (ρ-ϑ) space. The parameterization of the Hough transformation is based on the orthogonal distance ρ of the line to the origin and the direction ϑ of the normal to the line. Each pixel $p(x_{p},y_{p})$ forms a sinusoidal curve on the ρ-ϑ space:

(1) $$\rho =x_{p}\cos\vartheta +y_{p}\sin\vartheta .$$

Thus, a set of points that form a straight line will produce sinusoids which cross at the specific ρ-ϑ for that line. Therefore, finding collinear points on the image can be converted to the problem of finding accumulated peak in the ρ-ϑ space.

Short lines or falsely detected edges will significantly decrease the accuracy of line clustering and vanishing point calculation. That makes feature line detection and filtering indispensable. A voting scheme is used to select candidate peaks from the accumulated histogram for collinearity detection in ρ-ϑ space. For the best results, this study uses the inverted pyramid pattern iterative calculation for ρ-ϑ parameters, and the iteration stops when detected vanishing points are stable, as explained in Section 3.3.

### 3.3. Initial Vanishing Point Localization

According to the detected peaks in the first Hough transform, a second transformation for those peaks is employed to identify line segments passing through the same point (or small area) on the image. The local maximum peaks in the first Hough space appear to be collinear because that specific pixel (possible vanishing point) in the image contributes to all of the ρ-ϑ parameters’ accumulators.

Two conditions for obtaining stable vanishing points are considered. The first is the number of line segments in each direction, i.e., adjusting line group number threshold to prevent most detected lines from pointing to a certain direction. The other is that the representative vanishing points should be stable under different ρ-ϑ parameters. Modifying ρ-ϑ parameters can increase the reliability for vanishing point calculation.

Afterward, similarity rectification is applied to identify different line segments. Then, a least-squares method is employed to trace line groups interactively by adjusting the threshold of histogram peaks until the number of line groups is satisfied. Finally, vanishing points are calculated according to the grouped line segments and optimized with iterative calculation.

**Figure 2:**
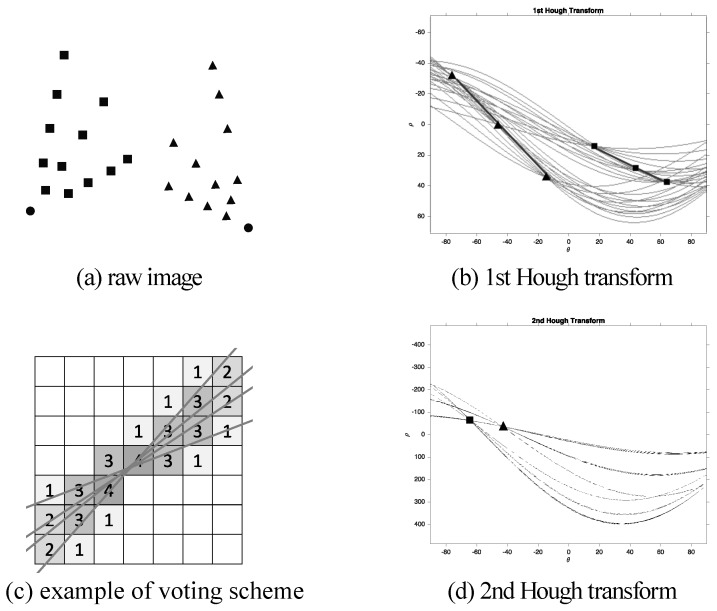
An example of a double Hough transform, (**a**) raw image: two group of dots are marked as rectangles and triangles; (**b**) first Hough transform finding the collinear points; (**c**) an example of voting scheme: the numbers of each accumulator represent how many lines are passing through it; (**d**) high peaks extracted from (**b**) are transformed as lines in the second Hough transform.

Figure 2a is an example of an input image. Two groups of dots are marked as rectangles and triangles, respectively. Each set of four points forms a line and the lines pass through two intersect points marked as dots. Figure 2b illustrates the result after the first Hough transformation, in which candidate peaks are marked as squares and triangles forming a line. An example of a voting scheme is shown in Figure 2c, and the number of each accumulator represents how many lines are passing through it. High peaks extracted from Figure 2b are transformed as lines in the second Hough transform as demonstrated in Figure 2d. The intersection of the lines are marked as points representing their groups in rectangles and triangles, respectively.

If necessary, a third Hough transform can be applied to the peaks of the second one to detect collinear vanishing points. These kinds of features can be used to construct vanishing lines.

## 4. Camera Calibration Using Vanishing Points

Vanishing point-based calibration is considered as one of the most practical calibration methods. The collinearity condition equations utilize the geometric position of a perspective center, an image point, and its corresponding object point as follows,

(2) $$\begin{array}{c} x_{p}=-f\left[\frac{M_{00}\left(X_{p}-X^{c}\right)+M_{01}\left(Y_{p}-Y^{c}\right)+M_{02}\left(Z_{p}-Z^{c}\right)}{M_{20}\left(X_{p}-X^{c}\right)+M_{21}\left(Y_{p}-Y^{c}\right)+M_{22}\left(Z_{p}-Z^{c}\right)}\right]+x_{0}\\ y_{p}=-f\left[\frac{M_{10}\left(X_{p}-X^{c}\right)+M_{11}\left(Y_{p}-Y^{c}\right)+M_{12}\left(Z_{p}-Z^{c}\right)}{M_{20}\left(X_{p}-X^{c}\right)+M_{21}\left(Y_{p}-Y^{c}\right)+M_{22}\left(Z_{p}-Z^{c}\right)}\right]+y_{0} \end{array},$$

where $x_{p}$ and $y_{p}$ are the image coordinates of a point; $X_{p},Y_{p},$ and $Z_{p}$ are the object space coordinates; $X^{c}$, $Y^{c}$, and $Z^{c}$ are the coordinates of the perspective center; *f* is the principal distance; $x_{0}$ and $y_{0}$ are the coordinates of principle point; and $M_{00}∼M_{22}$ are the elements of the 3 × 3 rotation matrix *M*, consisting of three rotation angles: ω (pan), ϕ (tilt), and κ (swing) [34]:

(3) $$\begin{array}{c} M=\left[\begin{array}{ccc} \cos\omega \cos\kappa +\sin\omega \sin\phi \sin\kappa & \sin\omega \cos\kappa -\cos\omega \sin\phi \sin\kappa & \cos\phi \sin\kappa \\ \sin\omega \sin\phi \cos\kappa -\cos\omega \sin\kappa & -\cos\omega \sin\phi \cos\kappa -\sin\omega \sin\kappa & \cos\phi \cos\kappa \\ -\sin\omega \cos\phi & \cos\omega \cos\phi & \sin\phi \end{array}\right] \end{array}.$$

**Figure 3:**
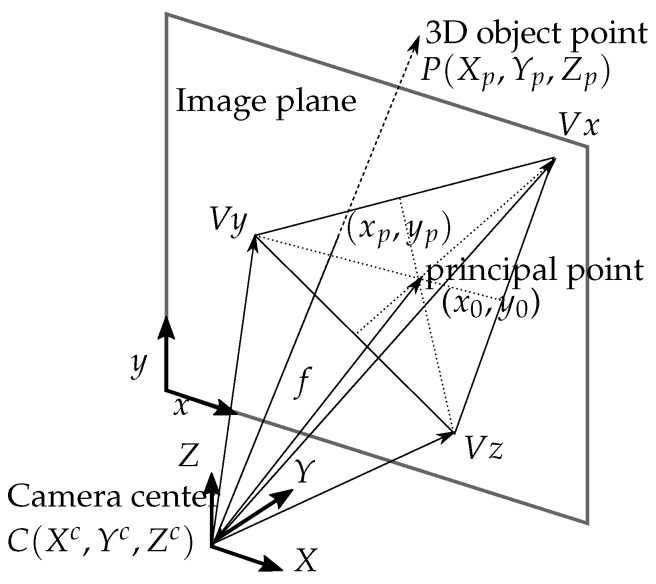
An illustration of the camera (exposure station), 3D point (object), and its photo image, which all lie on a straight line.

The camera and object coordinate systems are illustrated with geometric relations between vanishing points, the image plane, and the center of the camera in Figure 3.

Under the assumption of well-calibrated lens distortion of the perspective system, $f,x_{0}$, and $y_{0}$, and the three rotation angles ω,ϕ, and κ can be estimated using collinearity condition equations and three mutually orthogonal vanishing points. Vx,Vy, and Vz are the three vanishing points intersected from the parallel lines along the “X, Y, Z” axes in the object space, respectively [35]. Therefore, one can assume the vanishing points are at the infinity place in the object space. For example, at $X_{Vx}\approx \infty$, the vanishing point $Vx(x_{Vx},y_{Vx})$ following Equation (2) can be rewritten as:

(4) $$\begin{array}{cc} x_{Vx} & =-f\left[\frac{M_{00}\left(X_{Vx}-X^{c}\right)+M_{01}\left(Y_{Vx}-Y^{c}\right)+M_{02}\left(Z_{Vx}-Z^{c}\right)}{M_{20}\left(X_{Vx}-X^{c}\right)+M_{21}\left(Y_{Vx}-Y^{c}\right)+M_{22}\left(Z_{Vx}-Z^{c}\right)}\right]+x_{0}\\ & =-f\left[\frac{M_{00}\left(\frac{X_{Vx}}{X_{Vx}}-\frac{X^{c}}{X_{Vx}}\right)+M_{01}\left(\frac{Y_{Vx}}{X_{Vx}}-\frac{Y^{c}}{X_{Vx}}\right)+M_{02}\left(\frac{Z_{Vx}}{X_{Vx}}-\frac{Z^{c}}{X_{Vx}}\right)}{M_{20}\left(\frac{X_{Vx}}{X_{Vx}}-\frac{X^{c}}{X_{Vx}}\right)+M_{21}\left(\frac{Y_{Vx}}{X_{Vx}}-\frac{Y^{c}}{X_{Vx}}\right)+M_{22}\left(\frac{Z_{Vx}}{X_{Vx}}-\frac{Z^{c}}{X_{Vx}}\right)}\right]+x_{0}\\ & =-f\left[\frac{M_{00}}{M_{20}}\right]+x_{0}=f\frac{-\cos\omega \cos\kappa -\sin\omega \sin\phi \sin\kappa }{\sin\omega \cos\phi }+x_{0} \end{array},$$

(5) $$\begin{array}{cc} y_{Vx} & =-f\left[\frac{M_{10}}{M_{20}}\right]+y_{0}=f\frac{\cos\omega \sin\kappa -\sin\omega \sin\phi \cos\kappa }{\sin\omega \cos\phi }+y_{0} \end{array}.$$

Similarly, assuming $Y_{Vy}\approx \infty$ and $Z_{Vz}\approx \infty$, $Vy(x_{Vy},y_{Vy})$ and $Vz(x_{Vz},y_{Vz})$ can be derived as

(6) $$\begin{array}{cc} x_{Vy} & =-f\left[\frac{M_{01}}{M_{21}}\right]+x_{0}=f\frac{\sin\omega \cos\kappa -\cos\omega \sin\phi \sin\kappa }{\cos\omega \cos\phi }+x_{0}\\ y_{Vy} & =-f\left[\frac{M_{11}}{M_{21}}\right]+y_{0}=f\frac{-\sin\omega \sin\kappa -\cos\omega \sin\phi \cos\kappa }{\cos\omega \cos\phi }+y_{0} \end{array},$$

(7) $$\begin{array}{cc} x_{Vz} & =-f\left[\frac{M_{02}}{M_{22}}\right]+x_{0}=f\frac{\cos\phi \sin\kappa }{\sin\phi }+x_{0}\\ y_{Vz} & =-f\left[\frac{M_{12}}{M_{22}}\right]+y_{0}=f\frac{\cos\phi \cos\kappa }{\sin\phi }+y_{0} \end{array}.$$

From Equation (4) to Equation (7), the three vanishing points are only related to $f,x_{0}$, and $y_{0}$ and the three rotation angles ω, ϕ, and κ. These six unknowns can be solved using $Vx(x_{Vx},y_{Vx})$, $Vy(x_{Vy},y_{Vy})$ and $Vz(x_{Vz},y_{Vz})$.

### 4.1. Camera Orientation Calibration

A pair of vanishing points can be used to define a vector (vanishing line), and thus, three vectors can be found from the combination of three vanishing points:

(8) $$\left\{\begin{array}{c} {\vec{L}}_{VxVy}=\left(x_{Vy}-x_{Vx},y_{Vy}-y_{Vx}\right)\\ {\vec{L}}_{VyVz}=\left(x_{Vz}-x_{Vy},y_{Vz}-y_{Vy}\right)\\ {\vec{L}}_{VzVx}=\left(x_{Vx}-x_{Vz},y_{Vx}-y_{Vz}\right) \end{array}.\right.$$

The slope (*m*) of these three vanishing lines can be calculated as

(9) $$m_{VxVy}=\frac{y_{Vy}-y_{Vx}}{x_{Vy}-x_{Vx}}=\frac{-\sin\kappa }{\cos\kappa },$$

(10) $$m_{VyVz}=\frac{y_{Vz}-y_{Vy}}{x_{Vz}-x_{Vy}}=\frac{\cos\omega \cos\kappa +\sin\omega \sin\phi \sin\kappa }{\cos\omega \sin\kappa -\sin\omega \sin\phi \cos\kappa },$$

(11) $$m_{VzVx}=\frac{y_{Vx}-y_{Vz}}{x_{Vx}-x_{Vz}}=\frac{-\sin\omega \cos\kappa +\cos\omega \sin\phi \sin\kappa }{-\sin\omega \sin\kappa -\cos\omega \sin\phi \cos\kappa }.$$

Hence, κ can be determined from $m_{VxVy}$ as shown in Equation (9).

Angle ω and ϕ are therefore estimated from the multiplication and division of Equations (10) and (11), rewritten as Equations (12) and (13), respectively.

(12) $$\begin{array}{cc} \sin\phi^{2} & =\frac{\left(m_{VyVz}\sin\kappa -\cos\kappa \right)\left(-m_{VzVx}\sin\kappa +\cos\kappa \right)}{\left(m_{VyVz}\cos\kappa +\sin\kappa \right)\left(m_{VzVx}\cos\kappa +\sin\kappa \right)} \end{array},$$

(13) $$\begin{array}{cc} \tan^{2}\omega & =\frac{\left(m_{VyVz}\sin\kappa -\cos\kappa \right)\left(m_{VzVx}\cos\kappa +\sin\kappa \right)}{\left(-m_{VzVx}\sin\kappa +\cos\kappa \right)\left(m_{VyVz}\cos\kappa +\sin\kappa \right)} \end{array}.$$

### 4.2. Camera IOP Calibration

Each vanishing point and the principle point can also be used to define a vector, therefore, three vectors can be found from three vanishing points:

(14) $$\left\{\begin{array}{c} {\vec{L}}_{OVy}=\left(x_{Vy}-x_{O},y_{Vy}-y_{O}\right)\\ {\vec{L}}_{OVz}=\left(x_{Vz}-x_{O},y_{Vz}-y_{O}\right)\\ {\vec{L}}_{OVx}=\left(x_{Vx}-x_{O},y_{Vx}-y_{O}\right) \end{array}\right..$$

The orthocenter of the triangle formed from the three vanishing points of the three mutually orthogonal directions identifies the principal point through the inner product of the segments of the triangle and its heights. For instance, the inner product of ${\vec{L}}_{VyVz}$ and ${\vec{L}}_{OVx}$ is equal to zero due to the perpendicularity, and is same as the inner product of ${\vec{L}}_{VzVx}$ and ${\vec{L}}_{OVy}$. Hence, the principal point can be solved by expanding these two simultaneous equations.

The focal length, *f*, can be computed afterwards as the square root of the product of the distances from the principal point to any of the triangle’s vertices and the opposite side:

(15) $$\begin{array}{cc} Area & =\left|\frac{1}{2}\left|\begin{array}{ccc} x_{Vx} & y_{Vx} & 1\\ x_{Vy} & y_{Vy} & 1\\ x_{Vz} & y_{Vz} & 1 \end{array}\right|\right|=\left|\frac{1}{2}\left(\frac{f^{2}}{\sin\omega \cos\omega \sin\phi \cos^{2}\phi }\right)\right|, \end{array}$$

(16) $$f=\sqrt{2Area\cdot \left|\left(\sin\omega \cos\omega \sin\phi \cos^{2}\phi \right)\right|}.$$

According to the derivation above, the standard procedure of the three vanishing point-based camera calibration starts from the rotation angle estimation (Equations (9)–(11)), followed by principle point calculation, and finally, the focal length from Equation (16). Six unknowns thus can be solved with a unique solution.

If and only if the image is un-cropped and captured by a pinhole camera, the lens distortion calibration can also be achieved using vanishing points. The most commonly encountered lens distortion is radial distortion, including barrel and pincushion distortion. The standard model is formulated as:

(17) $$\begin{array}{c} x=x_{d}+k_{1}\left(x_{d}-x_{o}\right)r_{d}^{2}+k_{2}\left(x_{d}-x_{o}\right)r_{d}^{4}\;\\ y=y_{d}+k_{1}\left(y_{d}-y_{o}\right)r_{d}^{2}+k_{2}\left(y_{d}-y_{o}\right)r_{d}^{4}\;\\ r_{d}=\sqrt{{\left(x_{d}-x_{o}\right)}^{2}+{\left(y_{d}-y_{o}\right)}^{2}} \end{array},$$

where $x_{d}$ and $y_{d}$ are the corresponding image coordinates with distortion; $k_{1}$, and $k_{2}$ are the coefficients of radial distortion, and $r_{d}$ is the distorted radius.

To find the distortion parameters $k_{1}$ and $k_{2}$, this study follows the fundamental property of the perspective camera model. Vanishing points provide an useful constraint to estimate the radial distortion parameters using the line-fitting adjustment of an image point observed from the corresponding vanishing point. The observed image lines are constrained to converge to their corresponding vanishing point $V(x_{V},y_{V})$ according to the following equation:

(18) $$\left(x-x_{V}\right)\cos\theta +\left(y-y_{V}\right)\sin\theta =0.$$

Include the symmetric radial distortion parameters $k_{1}$ and $k_{2}$ into Equation (18), and it becomes:

(19) $$\begin{array}{c} (x_{d}+\left(x_{d}-x_{o}\right)\left(k_{1}\left(x_{d}^{2}-y_{d}^{2}\right)+k_{2}{\left(x_{d}^{2}-y_{d}^{2}\right)}^{2}\right)-x_{V})\cos\theta +\;\\ (y_{d}+\left(y_{d}-y_{o}\right)\left(k_{1}\left(x_{d}^{2}-y_{d}^{2}\right)+k_{2}{\left(x_{d}^{2}-y_{d}^{2}\right)}^{2}\right)-y_{V})\sin\theta =0 \end{array}.$$

When $x_{o}$ and $y_{o}$ are obtained, the line best-fit parameters of $k_{1}$ and $k_{2}$ can be estimated using a least median of squares (LMedS) procedure.

## 5. Vanishing Point Refinement

The vanishing points are imaginary points an infinite distance away from the projection center. Therefore, no direct measurement can be achieved to locate the exact locations of the vanishing points. It is difficult to extract vanishing points without random or systematic errors, especially in the cases of images with weak perspective geometry (e.g., long focal length). Consequently, increasing the reliability of the vanishing points’ positions is an important task for vanishing point-based camera calibration. The proposed vanishing point refinement process described here minimizes both random and systematic errors based on the constraints derived from common geometric properties of man-made structures. For instance, feature points pertaining to the same (flat) roof, building base, or floor etc. should have the same height or planar coordinates, however, biases may occur because of computational errors. The systematic error from the perspective of projection consistency thus provides an indication for fine-tuning the best positions of the vanishing points.

### 5.1. Feature Point Selection and Base Point Estimation

To estimate the perspective projection consistency using vanishing points, it is necessary to find sufficient feature points and their corresponding base points on the reference plane. The most common features of artificial structures are corner points at the intersected edges, planes, or boundaries. Thus, detection of feature points from the extracted long edges is more reliable than from the raw image. Short segments and small closed polygons from detected segments can be ignored because most of them are windows, patterns, or minor structures. The candidate feature points are then detected using Harris corner detector [36]. Some of the geometry constraints can be used for filtering feature point candidates. The first task is to define a reference plane with a reference origin and vanishing points along the X and Y axes, where origin (*O*) is normally formulated as,

(20) $$O=\frac{V_{x}+V_{y}+V_{z}}{3}.$$

**Figure 4:**
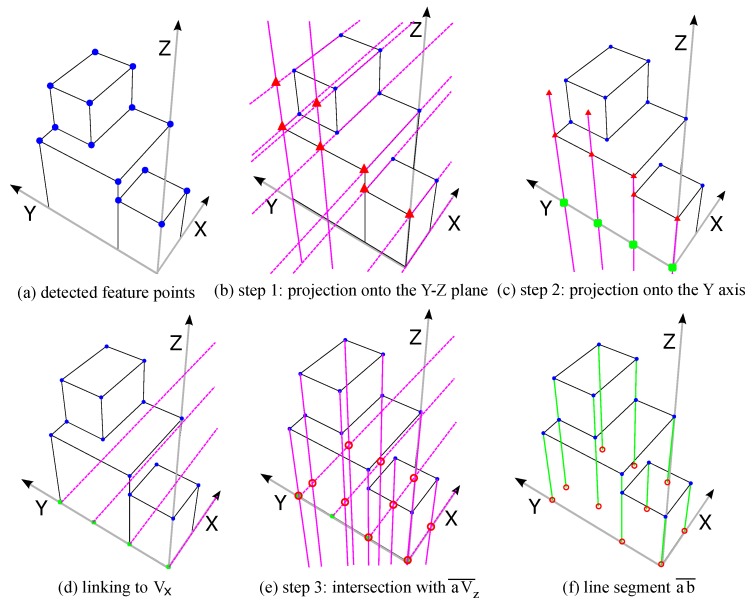
An example procedure of base point prediction. (**a**) Detected feature points (blue dots). (**b**) According to the $V_{x}$ and $V_{z}$, feature points are projected onto the Y-Z plane. The projections for the feature points are marked in red triangles. (**c**) $V_{z}$ is used to project red triangles onto the Y axis (green squares). (**d**) Green squares are recorded and linked to $V_{x}$. (**e**) The intersection points of the lines linked from feature points to $V_{z}$ are the base points (red points) corresponding to their feature points. (**f**) Green lines represent the heights between the feature points and base points.

The reference origin is defined as the intersection point from the bottom edges along the X and Y axes of the main structure. Candidate feature points below the reference plane or collinear to others can also be removed. However, the proposed procedure requires user interaction to make the final selection. The next task is to estimate base points. A base point is the vertical projection of a feature point onto the reference plane. It is a necessary element to estimate the consistency of perspective projection constructed from vanishing points. However, most base points are hidden in the image because of self-occlusion; only a few of them may have the potential to be extracted directly from the raw image. For estimating the corresponding base points, the proposed process is based on the characteristics of vanishing point constraints, and is an automatic and robust solution. Figure 4 illustrates a procedure for predicting base points. The extracted feature points are marked in round blue dots in Figure 4a. Following the assumption of the collinearity of the feature point (a) and the base point (b), the search area can be one-dimensional along the $\bar{aV_{z}}$. The task now is simplified into finding the horizontal location of the base point on $\bar{aV_{z}}$.

The estimation process can be generalized into three steps. First, all feature points are projected onto the Y–Z plane according to $V_{x}$ and $V_{z}$. The projected feature points are marked in red triangles as illustrated in Figure 4b. Points with the same height level and the same Y coordinate should overlap at the same projection point. Similarly, feature points can also be projected onto the X-Z plane using $V_{y}$ and $V_{z}$. Secondly, the red triangles can be further projected onto the Y or X axis, as noted in green squares in Figure 4c along $V_{z}$ to locate the Y coordinate of each feature point. Finally, candidate positions of the base points located on the line are linked from green squares to $V_{x}$ (or $V_{y}$) as shown in Figure 4d defining the horizontal locations of the base points. The intersection points to the lines in Figure 4d and $\bar{aV_{z}}$ are the estimated locations of the base points (red circles in Figure 4e) that are corresponding to feature points according to the path record. In Figure 4f, the green lines represent the target heights between the feature points and their corresponding base points which will be determined in the following procedure.

### 5.2. Vanishing Point Fine-Tuning

**Figure 5:**
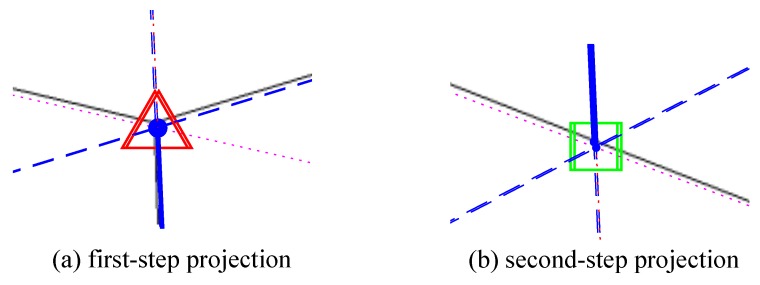
Demonstration of projection error during the base point searching process, (**a**) triangles are the first projection points on the Y–Z plane; (**b**) squares are the second projection points on the Y axis.

The error of each set of grouped projection point during the base points estimation process can be minimized by fine-tuning the positions of vanishing points. The vanishing point localization errors will cause the displacement during the projection process; therefore, the calibration results normally include systematic errors. Feature points with the same height and Y coordinate should be perfectly projected onto the same projection point as shown in Figure 5a, and points with the same Y coordinates should also overlap with the same position marked in Figure 5b.

The divergences in the first and second projection steps provide useful information for vanishing point refinement. The more precisely the vanishing point positions are estimated, the fewer the divergences that may occur during the projection process.

**Figure 6:**
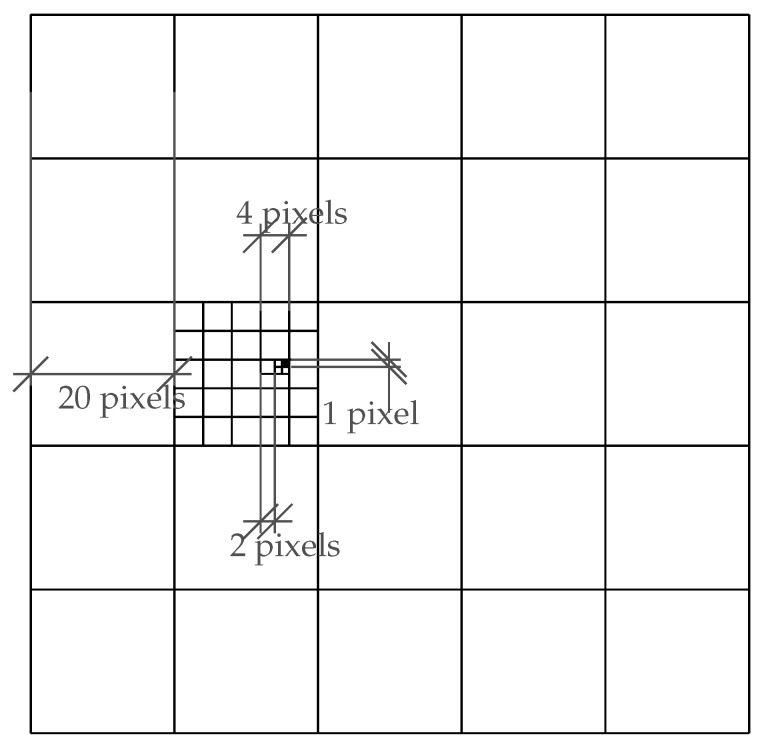
An example of the coarse-to-fine vanishing point fine-tuning process. The initial vanishing point is in the center. Every iteration reduces the span value and zooms in to a smaller area which has the lowest standard deviation (Equation (21)). This case reduced the calculation iteration from $O(n^{2})$(10,000) to O(1)(58), significantly increasing the processing efficiency.

To decide the fine-tuning values and orders, a moving pixel pyramid and half-and-half adjustment strategy was developed in the proposed algorithm. The objective is to minimize the standard deviation of each of the clustered projection points,

(21) $$\min\sum_{i,j}{\left(\dot{a}-\overset{\bar{˙}}{a}\right)}_{k},$$

where i,j are the fine-tuning pixels in the image space for each vanishing point; and $\dot{a}$ is the projected feature point in each step that belongs to group *k* with a mean value of $\overset{\bar{˙}}{a}$. Every fine-tuned pixel will update the standard deviation for each group. However, the traditional moving pixel approach takes $O(N^{2})$ interations for each vanishing point, where *N* is the number of moving pixels along horizontal and vertical axes. The proposed moving pixel pyramid is a coarse-to-fine approach, fine-tuning the vanishing points from large pixel spans to the sub-pixel level with O(1) computational complexity. The fine-tuning begins with larger pixel spans to locate a coarse area with the lowest standard deviation value. Then, the span pixel value is reduced to zoom in to a smaller area, until the iteration ends with the sub-pixel leveled fine-tuning. Figure 6 demonstrates an example of the pyramid fine-tuning approach, in which the initial vanishing point is in the center, and the searching boundary is from −50 to +50 pixels on both directions from top-left [−50,−50] to bottom-right [50,50]. The first fine-tuning span is of 20 pixels. After estimation and update of the vanishing point position 25 times, the process zooms in to the next level with a span value of four pixels. The same procedure continues and the vanishing point can be fine-tuned to an ideal position after $5^{2}+5^{2}+2^{2}+2^{2}=58$ iterations instead of $100^{2}=10,000$ iterations using the traditional pixel-by-pixel searching approach.

The fine-tuning process is based on statistic estimations, and it is difficult to determine which vanishing point localization displacement affects the overall errors most. In case of weak perspective geometry, one of the vanishing points may contain larger error than the others, but the fine-tuning process may reduce the error caused by the vanishing point which should not be adjusted. The proposed half-and-half adjustment strategy can reduce the error caused by a large vanishing point displacement. This strategy first adjusts the half-distance of each vanishing point from the original position to the optimized position. Which adjustment provides the greatest contribution should be determined and then that specific vanishing point should be fully adjusted towards the optimized position.

Geometrically, modifying $V_{x}$ and $V_{y}$ along the vertical direction in image space changes the vanishing line slope. Varying the slope of a vanishing line means the adjustment of the reference plane for optimizing all feature points perpendicular to it. An incorrect slope of the vanishing line estimation will cause a tapering effect shrinking to one side of the vanishing point and enlarging on the other side to the line segments that should have the same length. The horizontal change of $V_{x}$ and $V_{y}$ will resize the area formed by three vanishing points, which refers to the focal length calibration as mentioned in Equation (16).

## 6. Results and Discussion

**Figure 7:**
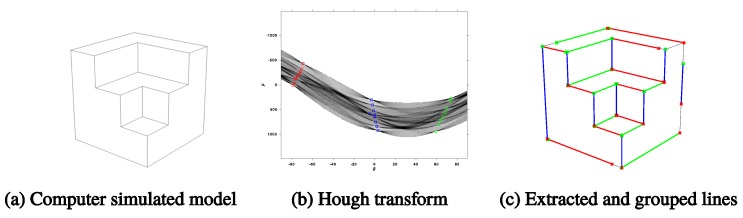
Line detection and grouping using the proposed cascade Hough transform. (**a**) Output image; (**b**) High peaks (squares) in the first Hough transform are grouped in the second Hough transform; (**c**) Three groups of lines are marked with green, yellow, and red, respectively.

A computer-simulated model test case (Figure 7a) was used to demonstrate the developed vanishing point estimation and refinement process step by step. The simulation image was generated using Trimble Sketch Up software. After creating the 3D model, it was output to an image with the perspective matrix projection camera module. Strong edge pixels were extracted from the raw image and converted into first Hough space for line detection. The high peaks in the first Hough transform were extracted with local maximum suppression, removing duplicated candidates due to over-segmentation. The extracted peaks represent the line equations in the image space. To clarify which lines belong to which vanishing point, the high peaks are further transformed in the second Hough space. The transformed peaks intersect at the same bin if they belong to the same vanishing point in the image space. The classified result (Figure 7c) consists of three groups of detected line equations marked with different colors.

**Figure 8:**
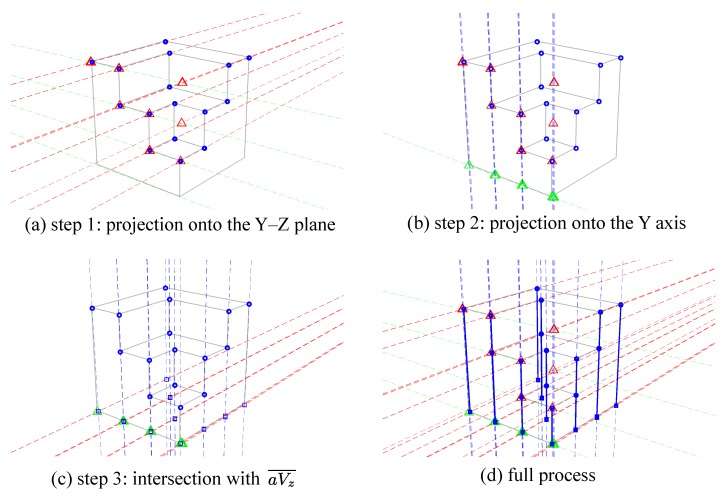
Base point estimation. (**a**) Blue feature points projected onto the Y−Z plane are marked as red triangles; (**b**) Red triangles projected onto the *Y* axis are marked as green triangles; (**c**) Base points are estimated from the intersection of $\bar{aV_{z}}$ and lines linked from green triangles to $V_{x}$; (**d**) The solid blue lines are $\bar{ab}$ with unknown height *h*.

The initial vanishing points are then used with selected feature points for searching for corresponding base points. A step-by-step base point estimation process is illustrated in Figure 8. All selected blue feature points are first projected onto the Y−Z plane from $V_{x}$, marked as red triangles (Figure 8a). Those red triangles are further projected to the direction of $V_{z}$ onto the *Y* axis, marked as green triangles (Figure 8b). Finally, the intersection of $\bar{aV_{z}}$ and lines linked from green triangles to $V_{x}$ indicate the base points (Figure 8c).

**Figure 9:**
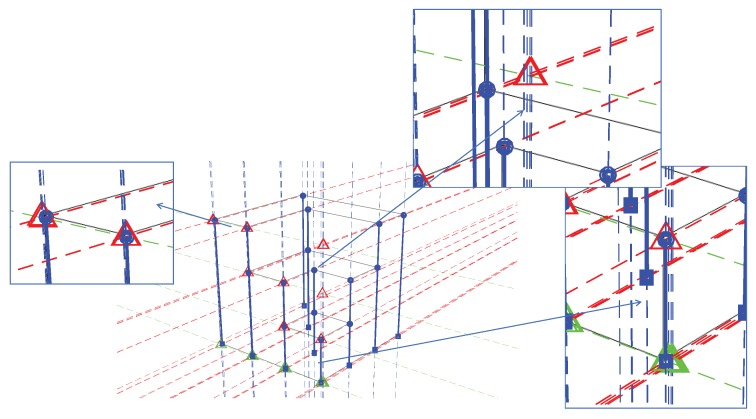
Base point estimation errors in each of the projection steps. The proposed vanishing point refinement process is designed to reduce the misalignment.

Figure 9 displays three enlarged parts of the projection process from Figure 8d. Several projection lines passing through $V_{z}$ are not well-overlapped. There appears to be a systematic misalignment due to vanishing point displacements.

**Figure 10:**
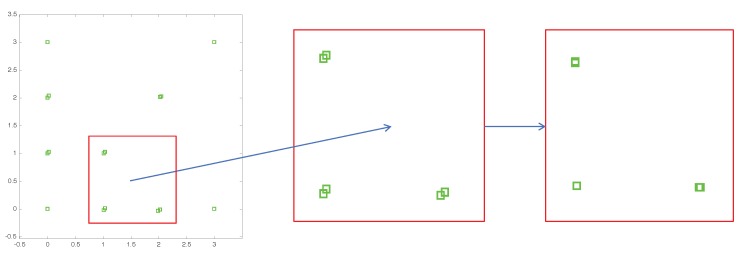
Base point estimation errors and fine-tuned results. Green squares on the left are the estimated base points. Several points should have been identically overlapped but are slightly dispersed (enlarged in the center). After vanishing point refinement, the divergences are reduced substantially (right figure).

Figure 10 demonstrates the estimated base points located on the referenced plane. Several points should have been identically overlapped on the same coordinates. However, the error during the initial vanishing point estimation caused the projecting errors in each projection step.

**Figure 11:**
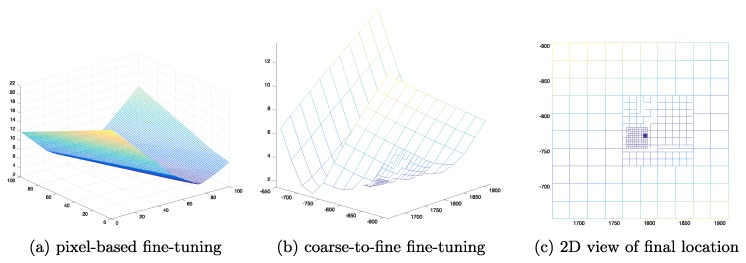
A coarse-to-fine fine-tuning comparison. The vertical axis shows the accumulated error value. Grid color from warm to cold represents the error from high to low. (**a**) Traditional pixel-based fine-tuning, covering −50∼50 pixels on the row and column with $100^{2}$ calculation times; (**b**) The proposed coarse-to-fine approach, covering −250∼250 pixels on the row and column with $4\times 10^{2}$ calculation times; (**c**) A two-dimensional (2D) view of each searching layer.

The proposed fine-tuning algorithm was applied to reduce the divergences. Figure 11 compares the conventional moving pixel-based (Figure 11a) and the proposed coarse-to-fine approaches (Figure 11b,c). This test case used a 10 × 10 coarse-to-fine fine-tuning moving pixel pyramid. The sampling spans were of 25, 10, 3, and 0.5 pixels, respectively for each level.

The fine-tuning is based on the statistic value from local (red triangles) or global (green triangles) divergences. Reducing the standard deviation for red triangle groups will decrease the local error of each point group. Minimizing the standard deviation for red triangles, however, may increase the global error. For instance, two red triangle groups at different heights should be in the same green triangle group. Fine-tuning the vanishing points may lead to divergences when projecting these two red triangle groups on the *Y* axis.

**Figure 12:**
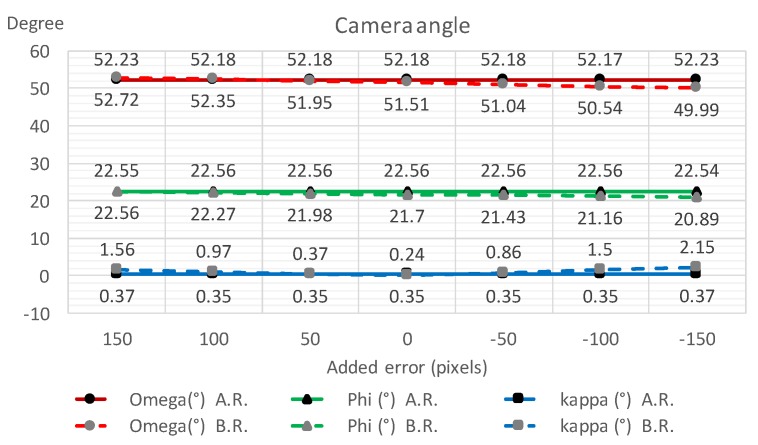
Camera angle estimation with and without the vanishing point refinement process with different manually added errors on $V_{x}$.

**Figure 13:**
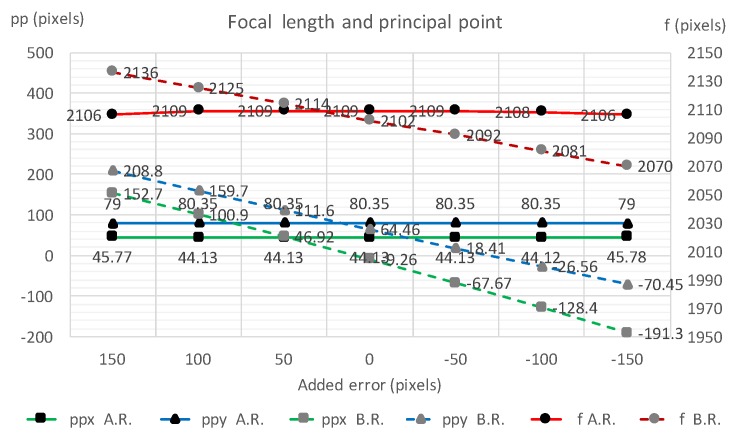
Camera principle point and focal length estimation with and without the vanishing point refinement process with different manually added errors on $V_{x}$.

**Figure 14:**
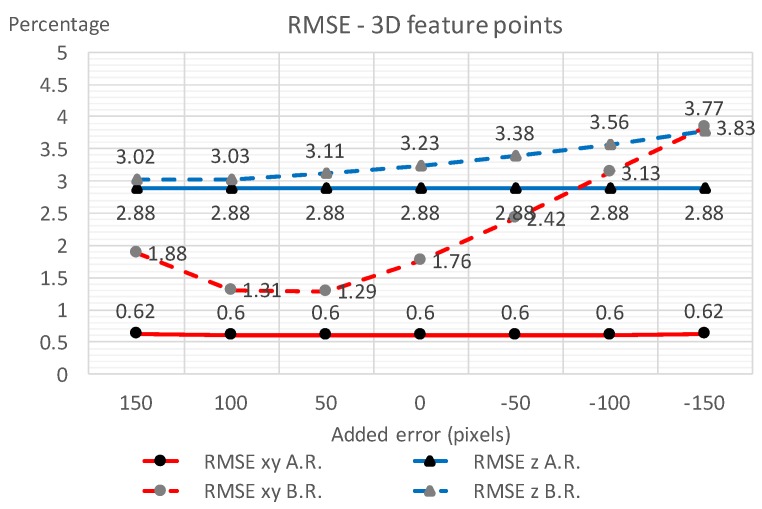
Three-dimensional (3D) point estimation with and without the vanishing point refinement process with different manually added errors on $V_{x}$.

To validate the robustness of the proposed refinement process, IOPs and EOPs were calculated with several additional offset errors manually put on to the initial vanishing point $V_{x}$ (Table 1). The listed results show that the EOP differences are of less than $0.1^{\circ }$ and the IOP differences are less than 3 pixels—a significant decrease in errors and a consistent improvement of the 3D point measurement accuracy. Figure 12, Figure 13 and Figure 14 display the robustness of the refinement process, demonstrating that the proposed refinement is capable of reducing the uncertainty of the initial vanishing point estimation.

**Figure 15:**
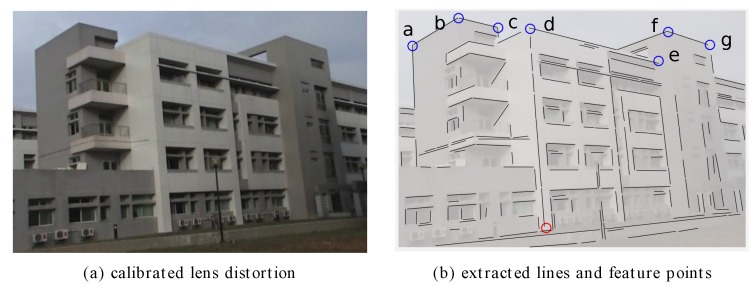
A test case of a video frame cut of a real building; the resolution is of 704 × 480 pixels. (**a**) A video frame calibrated using straight line segments; (**b**) Extracted lines and feature points for vanishing point estimation and 3D modeling.

Figure 15 was extracted from a video sequence with a dimension of 704 × 480 pixels in JPEG format. The estimated radial distortion coefficients $k_{1}$ and $k_{2}$ are $-2.267^{-7}$ and $1.273^{-12}$, respectively, calibrated using straight line segments. Figure 15b shows the extracted lines with feature points (*a* to *g*) of the targeted building. This case assumed the back side of the buildings have the same X−Y coordinates as the targeted structure on the left (Feature *a*, *b* and *c*).

**Figure 16:**
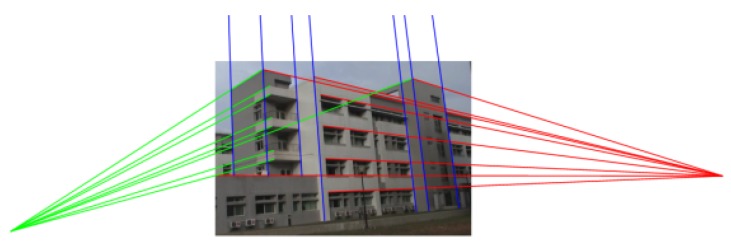
An illustration of three orthogonal vanishing points detected from the video frame cut; vanishing points linked to grouped line segments along X,Y,Z direction are marked in red, green, and blue, respectively.

Classified line segments are then used to estimate the initial location of the vanishing points. Because the tilt angel is low in this case, the vanishing point in the vertical (*Z*) direction is far away from the center (Figure 16). Table 2 lists the calibrated camera parameters with and without the proposed vanishing point refinement process using both a raw image and a lens distortion-calibrated image.

The reconstructed model was compared with field-surveyed data for quantitative analysis of the accuracy (Table 3), which also evaluated accuracy improvement of the building height estimation after the refinement of vanishing points. Using the measured distance (17.4 m) between feature point *d* and its corresponding base point as the reference, the maximum error of feature point *f* is about 3%. After the vanishing point refinement, not only were feature points of the same level correctly assigned with identical heights, but the overall RMSE decreased to less than 0.7%. Validations of 3D point measurements (Table 4) were compared with field-surveyed data from tape and laser measurements.

## 7. Conclusions

This paper presented a novel camera calibration approach based on vanishing point geometry. The proposed algorithms can be used to obtain reliable camera parameters without prior information and are particularly useful for onsite camera calibration. The proposed algorithms can also deal with the uncertainty of vanishing point calculation, that may significantly affect the camera IOPs/EOPs estimation. The main contribution of this study is the proposed vanishing point refinement strategy, which can significantly reduce the systematic and random errors stemming from the vanishing point localization. The fine-tuning process can minimize the projection error of each feature point after a few iterations using the half–half adjustment. A coarse-to-fine fine-tuning approach is also proposed to improve the processing efficiency from $O(n^{2})$ to O(1). To extract and group line segments for initial vanishing point estimation, this study improved cascade Hough transformation with adaptive thresholds. Extracted line segments are more robustly classified to the corresponding vanishing point.

Experiment results shown in this paper also demonstrate the robustness of the proposed refinement approach under high initial vanishing point estimation errors, improving 3D point reconstruction accuracy by 30% and keeping the estimated camera parameters consistent under additional vanishing point localization errors. A video frame case evaluated the improvement of the proposed vanishing point refinement process. The height measurement error was reduced from 2.04% to 0.64%. The proposed algorithms can be implemented on in situ camera calibration, single view metrology, and simultaneous localization and mapping (SLAM) applications in the human-made environment. Future improvements will focus on the integration into the SLAM system as a real-time camera pose tracking attribute. The developed calibration strategy also has a great potential for implementation with panoramic and omnidirectional cameras.

## Figures and Tables

**Table 1:** The performance of the proposed vanishing point refinement algorithm. System error on $V_{x}$ was manually added to validate the reliability under different initial vanishing point conditions.

Added Error (Pixels)		Omega (${}^{\circ }$)	Phi (${}^{\circ }$)	Kappa (${}^{\circ }$)	f (Pixels)	$x_{0}$ (Pixels)	$y_{0}$ (pixels)	RMSE_xy (%)	RMSE_z (%)
150	B.R.	52.72	22.56	1.56	2136	152.7	208.8	1.88	3.02
	A.R.	**52.23**	**22.55**	**0.37**	**2106**	**45.77**	**79**	**0.62**	**2.88**
100	B.R.	52.35	22.27	0.97	2125	100.9	159.7	1.31	3.03
	A.R.	**52.18**	**22.56**	**0.35**	**2109**	**44.13**	**80.35**	**0.6**	**2.88**
50	B.R.	51.95	21.98	0.37	2114	46.92	111.6	1.29	3.11
	A.R.	**52.18**	**22.56**	**0.35**	**2109**	**44.13**	**80.35**	**0.6**	**2.88**
0	B.R.	51.51	21.7	0.24	2102	−9.26	64.46	1.76	3.23
	A.R.	**52.18**	**22.56**	**0.35**	**2109**	**44.13**	**80.35**	**0.6**	**2.88**
−50	B.R.	51.04	21.43	0.86	2092	−67.67	18.41	2.42	3.38
	A.R.	**52.18**	**22.56**	**0.35**	**2109**	**44.13**	**80.35**	**0.6**	**2.88**
−100	B.R.	50.54	21.16	1.5	2081	−128.4	−26.56	3.13	3.56
	A.R.	**52.17**	**22.56**	**0.35**	**2108**	**44.12**	**80.35**	**0.6**	**2.88**
−150	B.R.	49.99	20.89	2.15	2070	−191.3	−70.45	3.83	3.77
	A.R.	**52.23**	**22.54**	**0.37**	**2106**	**45.78**	**79**	**0.62**	**2.88**

B.R.: before refinement; A.R.: after refinement.

**Table 2:** The comparison of interior orientation parameters (IOPs) and exterior orientation parameters (EOPs) before and after the proposed vanishing point refinement processing of the raw image and the radial distortion calibrated image.

	Un-Calibrated		Calibrated	
	B.R.	A.R.	B.R.	A.R.
Omega (${}^{\circ }$)	45.21	46.34	47.90	46.75
Phi (${}^{\circ }$)	12.11	12.36	12.39	12.58
kappa (${}^{\circ }$)	5.29	5.41	5.79	5.46
*f* (pixels)	1008	1017	1031	1047
$x_{0}$ (pixels)	2.99	9.12	43.02	16.32
$y_{0}$ (pixels)	−49.59	−55.41	−59.74	−57.51

B.R.: before refinement; A.R.: after refinement.

**Table 3:** Accuracy assessment of the height measurement of a video frame cut (unit: m).

Feature	Reference	Non-Refined	Residual	Error	Refined	Residual	Error
a	19.4	19.102	0.298	1.54%	19.387	0.013	0.07%
b	19.4	19.173	0.227	1.17%	19.387	0.013	0.07%
c	19.4	19.046	0.354	1.82%	19.387	0.013	0.07%
d*	17.4	–	–	–	–	–	–
e	17.4	17.173	0.227	1.30%	17.243	0.157	0.90%
f	20.5	21.136	−0.636	3.10%	20.686	−0.186	0.91%
g	20.5	21.022	−0.522	2.55%	20.686	−0.186	0.91%
RMSE			0.41	2.04%		0.13	0.64%

Feature d* is the reference height (17.4 m).

**Table 4:** The error analysis of 3D point measurement in the video frame cut test case (unit: m).

Feature	Reference			Proposed Method			Residual		
	X	Y	Z	X	Y	Z	X	Y	Z
a	−3.5	15.87	19.4	−3.42	15.4	19.39	−0.08	0.47	0.01
b	−3.5	6.33	19.4	−3.42	6.31	19.39	−0.08	0.02	0.01
c	0.5	6.33	19.4	0.52	6.31	19.39	−0.02	0.02	0.01
d*	0	0	17.4	–	–	–	–	–	–
e	18.25	0	17.4	18.54	0	17.24	−0.29	0	0.16
f	26.97	−1.5	20.5	27.46	−1.47	20.69	−0.49	−0.03	−0.19
g	26.97	−1.5	20.5	27.46	−1.47	20.69	−0.49	−0.03	−0.19
RMSE							0.21	0.19	0.13

Feature d* is the reference height (17.4 m).

## References

[1] Zhang Z. (2014). Camera calibration. Computer Vision.

[2] Tsai R. (1987). A versatile camera calibration technique for high-accuracy 3D machine vision metrology using off-the-shelf TV cameras and lenses. IEEE J. Robot. Autom..

[3] Zhang Z. (2000). A flexible new technique for camera calibration. IEEE Trans. Pattern Anal. Mach. Intell..

[4] Sturm P.F., Maybank S.J. On plane-based camera calibration: A general algorithm, singularities, applications. Proceedings of the 1999 IEEE Computer Society Conference on Computer Vision and Pattern Recognition.

[5] Caprile B., Torre V. (1990). Using vanishing points for camera calibration. Int. J. Comput. Vis..

[6] Gracie G. Analytical photogrammetry applied to single terrestrial photograph mensuration. Proceedings of the XIth International Congress of Photogrammetry.

[7] Chang H., Tsai F. (2012). Reconstructing Three-Dimensional Specific Curve Building Models from a Single Perspective View Image. Int. Arch. Photogramm. Remote Sens. Spat. Inf. Sci..

[8] Barnard S.T. (1983). Interpreting perspective images. Artif. Intell..

[9] Shufelt J.A. (1999). Performance evaluation and analysis of vanishing point detection techniques. IEEE Trans. Pattern Anal. Mach. Intell..

[10] Brauer-Burchardt C., Voss K. Robust vanishing point determination in noisy images. Proceedings of the 15th International Conference on Pattern Recognition.

[11] Kalantari M., Jung F., Guedon J. (2009). Precise, automatic and fast method for vanishing point detection. Photogramm. Record.

[12] Gonzalez-Aguilera D., Gomez-Lahoz J. (2008). From 2D to 3D through modelling based on a single image. Photogramm. Record.

[13] Bazin J.C., Pollefeys M. 3-line RANSAC for orthogonal vanishing point detection. Proceedings of the 2012 IEEE/RSJ International Conference on Intelligent Robots and Systems.

[14] Duda R.O., Hart P.E. (1972). Use of the Hough transformation to detect lines and curves in pictures. Commun. ACM.

[15] Lutton E., Maitre H., Lopez-Krahe J. (1994). Contribution to the determination of vanishing points using Hough transform. IEEE Trans. Pattern Anal. Mach. Intell..

[16] Gamba P., Mecocci A., Salvatore U. Vanishing point detection by a voting scheme. Proceedings of the International Conference on Image.

[17] Tuytelaars T., Proesmans M., Van Gool L. (1997). The cascaded Hough transform as support for grouping and finding vanishing points and lines. International Workshop on Algebraic Frames for the Perception-Action Cycle.

[18] Tuytelaars T., Van Gool L., Proesmans M., Moons T. The cascaded Hough transform as an aid in aerial image interpretation. Proceedings of the Sixth International Conference on Computer Vision.

[19] Tsai F., Chang H. Detection of Vanishing Points Using Hough Transform for Single View 3D Reconstruction. Proceedings of the 34th Asian Conference on Remote Sensing.

[20] De la Escalera A., Armingol J.M. (2010). Automatic Chessboard Detection for Intrinsic and Extrinsic Camera Parameter Calibration. Sensors.

[21] Matessi A., Lombardi L. (1999). Vanishing point detection in the hough transform space. European Conference on Parallel Processing.

[22] Cantoni V., Lombardi L., Porta M., Sicard N. Vanishing point detection: representation analysis and new approaches. Proceedings of the 11th International Conference on Image Analysis and Processing.

[23] Zhao Y.X., Tai H.P., Fang S.J., Chou C.H. A new validity measure and fuzzy clustering algorithm for vanishing-point detection. Proceedings of the International Conference on Automatic Control and Artificial Intelligence (ACAI 2012).

[24] McLean G., Kotturi D. (1995). Vanishing point detection by line clustering. IEEE Trans. Pattern Anal. Mach. Intell..

[25] Schaffalitzky F., Zisserman A. (2000). Planar grouping for automatic detection of vanishing lines and points. Image Vis. Comput..

[26] Almansa A., Desolneux A., Vamech S. (2003). Vanishing point detection without any a priori information. IEEE Trans. Pattern Anal. Mach. Intell..

[27] Desolneux A., Moisan L., Morel J.M. (2001). Edge detection by Helmholtz principle. J. Math. Imaging Vis..

[28] Košecká J., Zhang W. (2002). Video compass. European Conference on Computer Vision.

[29] Wildenauer H., Vincze M. Vanishing point detection in complex man-made worlds. Proceedings of the 14th International Conference on Image Analysis and Processing.

[30] Tardif J.P. Non-iterative approach for fast and accurate vanishing point detection. Proceedings of the IEEE 12th International Conference on Computer Vision.

[31] Choi J., Kim W., Kong H., Kim C. Real-time vanishing point detection using the Local Dominant Orientation Signature. Proceedings of the 3DTV Conference: The True Vision-Capture, Transmission and Display of 3D Video (3DTV-CON).

[32] Hartley R., Zisserman A. (2003). Multiple View Geometry in Computer Vision.

[33] Canny J. (1986). A computational approach to edge detection. IEEE Trans. Pattern Anal. Mach. Intell..

[34] Haralick R.M. (1980). Using perspective transformations in scene analysis. Comput. Graph. Image Process..

[35] Wang L.L., Tsai W.H. (1990). Computing camera parameters using vanishing-line information from a rectangular parallelepiped. Mach. Visi. Appl..

[36] Harris C., Stephens M. A combined corner and edge detector. Proceedings of the Alvey Vision Conference.

